# Complete mitochondrial genome of the Salt Creek pupfish, *Cyprinodon salinus salinus*: characterization and identification of single nucleotide polymorphisms (SNPs)

**DOI:** 10.1080/23802359.2021.1945964

**Published:** 2021-07-05

**Authors:** Ashley A. Del Core, Catie S. Cleveland, Sean C. Lema

**Affiliations:** Biological Sciences Department, California Polytechnic State University, San Luis Obispo, CA, USA

**Keywords:** Pupfish, mtDNA, Cyprinodontiformes, genetic variation, mitochondrial DNA

## Abstract

The Salt Creek pupfish, *Cyprinodon salinus salinus* Miller, 1943 is endemic to Death Valley, California, USA, and resides as a single population within one of the most extreme inland aquatic environments capable of supporting fish. Here we report the sequencing of complete 16,499 base pair (bp) mitochondrion genomes from four *C. salinus salinus* individuals. The mitochondrial genome of *C. salinus salinus* comprises 13 protein-coding regions, 12S and 16S rRNAs, 22 tRNAs, and an 832 bp D-loop region. The first reported single nucleotide polymorphisms (SNPs) were identified within the mtDNA of *C. salinus salinus*, with the four mitogenomes exhibiting only 0.0485% nucleotide sequence divergence indicative of low intraspecific variation. These complete mitogenomes will facilitate future genetic analyses of intraspecific diversity between the two described subspecies of *C. salinus* as well as other *Cyprinodon* pupfishes in southwestern North America.

The Salt Creek pupfish *Cyprinodon salinus salinus* Miller 1943 (Cyprinodontidae, Cyprinodontiformes) is one of several *Cyprinodon* pupfishes that evolved in the Death Valley region of eastern California and southwestern Nevada, USA (Miller 1948, 1950). Occurring within one of the driest and hottest regions in the world, aquatic environments in Death Valley stand out for their extreme conditions (Soltz and Naiman 1978). *Cyprindon salinus salinus* occupies one of the most extreme of these environments: Salt Creek, a small (∼1.5 km) perennial, spring-fed stream located approximately −40 to −68 m below sea level in Death Valley. Salt Creek has a consistently high salinity and temperatures that can exceed 40 °C during summer (Miller 1943; Jones 2017). The Salt Creek pupfish and other pupfishes of Death Valley evolved physiological abilities to survive such conditions (e.g. Heuton et al. 2015; Lema, Chow, et al. 2016), and *C. salinus* has been the focus of studies on osmoregulation under high salinities and temperatures (Naiman et al. 1976; Stuenkel and Hillyard 1980, 1981). Notably, *C. salinus salinus* is one of two subspecies of *C. salinus*, with the other subspecies – the Cottonball Marsh pupfish (*Cyprindon salinus milleri*, formerly *C. milleri*; La Bounty and Deacon 1972) – isolated to a hypersaline marsh into which Salt Creek may empty during years of extraordinary rainfall.

Here we report the first complete mitochondrial DNA genomes for the Salt Creek pupfish, *C. salinus salinus*. Complete mitogenomes were sequenced from two males (40.45 mm standard length [SL] and 1.28 g mass, 36.90 mm SL and 1.12 g) and two females (37.75 mm SL and 1.56 g; 33.45 mm SL and 0.74 g) collected on 27 February 2016 from Salt Creek (36.59138889 N, 116.99694444 W). DNA was extracted from skeletal muscle (DNeasy Blood & Tissue Kit, Qiagen, Valencia, CA, USA), and mtDNA was amplified (GoTaq^®^ Long PCR Master Mix, Promega Corp., Madison, WI, USA) using primers designed previously for sequencing the mitogenome of *Cyprinodon variegatus* (Barcelon and Lema 2016). PCR products were cleaned (QIAQuick PCR Purification Kit, Qiagen, Germantown, MD, USA), Sanger sequenced (MC Lab, South San Francisco, CA, USA), assembled using Sequencher v5.4.6 software (Gene Codes Corp., Ann Arbor, MI, USA), and annotated using MitoFish (Iwasaki et al. 2013; Sato et al. 2018).

The four sequenced mitochondrial genomes of *C. salinus salinus* (GenBank accession nos. MW446237, MW446238, MW446239, and MW446240) are each 16,499 bp in length and contain 13 protein-coding genes, 22 tRNAs, 12S and 16S rRNAs, and a D-loop region of 842 bp, which follows the mitogenome arrangement of other *Cyprinodon* pupfishes (Keepers et al. 2016; Barcelon and Lema 2016; Lema, Wilson, et al. 2016). Phylogenetic analysis substantiates *C. salinus salinus* as monophyletic with other Death Valley region pupfishes *Cyprinodon diabolis* (KX061747), *Cyprinodon nevadensis pectoralis* (KP064222), and *Cyprinodon nevadensis amargosae* (KU883631) (Figure 1). Notably, that phylogenetic analysis also revealed the White Sands pupfish (*Cyprinodon tularosa*) (KP013105) clustered between two independently sequenced *C. variegatus* mitogenomes, as was also observed by Lema, Wilson, et al. (2016). Recent unpublished nuclear DNA analyses suggest possible genetic introgression of *C. variegatus* into *C. tularosa* (Black et al. 2021), which may explain relationships of those taxa in Figure 1.

**Figure 1.**
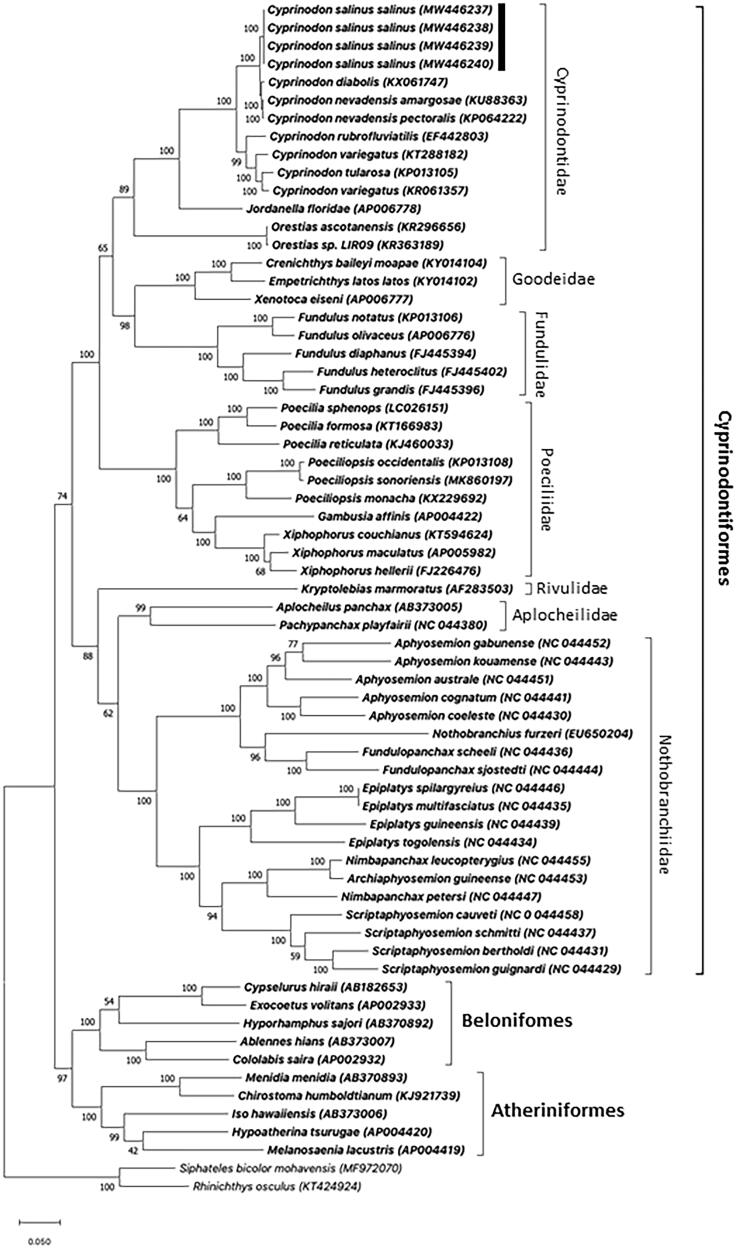
Maximum–likelihood phylogenetic tree derived from alignment of complete mitogenomes illustrates the taxonomic relationships of *Cyprinodon salinus salinus* (indicated by black bar) to other fishes of order Cyprinodontiformes, as well as select fishes of orders Beloniformes and Atheriniformes. Nucleotide sequences of complete mitogenomes were aligned using Clustal X (Larkin et al. 2007). Evolutionary history was first inferred using the Neighbor-joining method (Saitou and Nei 1987), and then tested via Maximum-likelihood with the Tamura–Nei substitution model. Bootstrap confidence values (1000 replicates) are indicated at each node. Phylogenetic relationships were constructed in MEGA X software (Kumar et al. 2018; Stecher et al. 2020). GenBank accession numbers accompany each taxon. Mitogenomes were complete for all taxa except *Jordanella floridae*, which lacked sequence for the D-loop region. The Mohave tui chub (*Siphateles bicolor mohavensis*) (Glaser et al. 2017) and speckled dace (*Rhinichthys osculus*) (Bock et al. 2016), two cyprinid fishes (order Cypriniformes) native to the same Mojave Desert region as the Salt Creek pupfish, were used as outgroups.

Comparison of the four *C. salinus salinus* mitogenomes revealed only 8 nucleotide sites of the entire 16,499 bp mitogenome varied among the specimens. That 0.0485% nucleotide sequence divergence is considerably less than the average 0.38% intraspecific mitogenome variation reported by Schroeter et al. (2020) in a survey of 65 fish species. A single nucleotide polymorphism (SNP) site was detected in the 12S (mitogenome location bp 418) and 16S (bp 2444) rRNAs of *C. salinus salinus*, as well as in tRNA-Gln (bp 3909). One SNP was also detected in each of five protein-coding genes; those SNPs encoded silent mutations in cytochrome c oxidase subunit 1 (*cox1,* bp 6432), cytochrome c oxidase subunit 3 (*cox3*, bp 9534), and NADH dehydrogenase subunit 4 (*nd4*, bp 11,069), but resulted in a Ser/Asn replacement in ND1 (mitogenome bp 3295) and Thr/Ala change in ND5 (bp 12,259). Previously, Echelle and Dowling (1992) did not find any variation in two *C. salinus salinus* specimens using restriction digestion of mtDNA. A separate study using 1047 bp of *nd2* and a 335 bp region of the D-loop similarly did not detect any variation among 28 specimens of *C. salinus salinus* (Duvernell and Turner 1998), which is consistent with the absence of SNPs in those regions of the mitogenomes characterized here. Nuclear DNA comparison of retrotransposon *swimmer 1* also found *C. salinus salinus* genetically monomorphic (Duvernell and Turner 1999). Our findings here are thus the first to document intraspecific genetic variation in the Salt Creek pupfish. Those SNPs may facilitate future studies of both fine-scale spatial movement and geographic variation in genetic diversity in *C. salinus salinus*.

## Data Availability

The mitochondrion genome sequence data that support the findings of this study are openly available in GenBank of the National Center for Biotechnology Information (NCBI) at https://www.ncbi.nlm.nih.gov/ under the accession nos. MW446237 to MW446240. Tissues from these specimens (Cat. nos. LACM T-001594 to T-001597) used for mitogenome sequencing were deposited into collections of the Department of Ichthyology at the Natural History Museum of Los Angeles County (https://nhm.org/research-collections/departments-and-programs/ichthyology; Dr. Todd Clardy, Collections Manager, tclardy@nhm.org).
